# Noninvasive Estimation of Hydration Status in Athletes Using Wearable Sensors and a Data-Driven Approach Based on Orthostatic Changes

**DOI:** 10.3390/s21134469

**Published:** 2021-06-29

**Authors:** Fahad Kamran, Victor C. Le, Adam Frischknecht, Jenna Wiens, Kathleen H. Sienko

**Affiliations:** 1 Division of Computer Science and Engineering, University of Michigan College of Engineering, Ann Arbor, MI 48109, USA; fhdkmrn@umich.edu (F.K.); wiensj@umich.edu (J.W.); 2 Department of Mechanical Engineering, University of Michigan College of Engineering, Ann Arbor, MI 48109, USA; victle@umich.edu; 3 Department of Pediatrics, Tanner Clinic, Layton, UT 84041, USA; adam.frischknecht@gmail.com

**Keywords:** dehydration, machine learning, dehydration, heart rate, exercise, orthostatic hypotension

## Abstract

Dehydration beyond 2% bodyweight loss should be monitored to reduce the risk of heat-related injuries during exercise. However, assessments of hydration in athletic settings can be limited in their accuracy and accessibility. In this study, we sought to develop a data-driven noninvasive approach to measure hydration status, leveraging wearable sensors and normal orthostatic movements. Twenty participants (10 males, 25.0 ± 6.6 years; 10 females, 27.8 ± 4.3 years) completed two exercise sessions in a heated environment: one session was completed without fluid replacement. Before and after exercise, participants performed 12 postural movements that varied in length (up to 2 min). Logistic regression models were trained to estimate dehydration status given their heart rate responses to these postural movements. The area under the receiver operating characteristic curve (AUROC) was used to parameterize the model’s discriminative ability. Models achieved an AUROC of 0.79 (IQR: 0.75, 0.91) when discriminating 2% bodyweight loss. The AUROC for the longer supine-to-stand postural movements and shorter toe-touches were similar (0.89, IQR: 0.89, 1.00). Shorter orthostatic tests achieved similar accuracy to clinical tests. The findings suggest that data from wearable sensors can be used to accurately estimate mild dehydration in athletes. In practice, this method may provide an additional measurement for early intervention of severe dehydration.

## 1. Introduction

Exercise-induced dehydration is typically a result of insufficient replenishment of fluids lost mainly to sweat. Dehydration of up to 2 to 3% of one’s body weight in athletic settings is common for healthy individuals, especially when performing in the heat [1]. Dehydration can predispose individuals to a variety of heat illnesses, including heat stroke and heat exhaustion [2,3,4,5]. Heat stroke for example, is the third leading cause of death in high school athletes and is regularly reported among other occupations that encounter heat stress [5].

To lower the potential risk of heat-related injuries, it is important to monitor hydration status and rehydrate during exercise [6,7,8] (e.g., drinking to thirst [9,10,11] or planned drinking programs to minimize bodyweight loss [6,7,10,12,13]. Laboratory-based approaches for monitoring dehydration status (e.g., serum chemistry panels), while accurate, require specialized equipment and can be difficult to administer during athletic activities due to inadequate portability [10,14,15]. Multiple types of wearable sensors have been used to estimate hydration status within research settings. For example, dehydration has been accurately estimated using sensors that analyze different biomarkers of sweat secretion or properties of the skin (e.g., temperature and impedance) that are correlated with hydration status [16,17,18]. Measuring changes in bodyweight is one of the most common means of assessing hydration status, primarily due to its simplicity and low cost [10,15,19]. However, in athletic settings, it is often difficult to accurately assess fluid loss without nude bodyweight measurements. Clothed bodyweight measurements may be rendered inaccurate over time due to sweat captured in clothes and other parts of the body [10,20,21,22]. Other confounders affecting bodyweight measurements include the time of day, respiratory water loss, and substrate oxidation [22,23]. Therefore, to improve hydration assessments during exercise, there is a need to develop complementary approaches to current field-based bodyweight measurements [23].

There exists a potential opportunity to leverage the data that are already collected in athletic contexts for estimating hydration status. Many professional and collegiate athletes are equipped with wearable technologies in the field that inform training and performance; examples of collegiate teams include Duke and University of Michigan Basketball. Devices like the Catapult OptimEye (catapultsports.com) can continuously collect data such as heart rate and position during games and practices [24,25,26]. The specific types of data and their accessibility create a promising opportunity for developing a noninvasive, complementary approach for assessing hydration status.

In this study, we explored a potential approach for using data from existing wearables to detect early levels of dehydration. In particular, we explored wearable devices that measured heart rate and postural orientation over an extended period of time. Using these data, we leveraged the relationship between hydration and cardiovascular responses to orthostatic changes for assessing hydration status: when an individual is dehydrated, heart rate increases significantly as part of an overall compensatory response to a decreased cardiovascular return due to orthostatic changes [27,28].

Previous work has investigated orthostatic movements to measure exercise-induced dehydration. However, prior work only used summary statistics (e.g., peak heart rate response) [29], considered the delayed effects of exercise-induced dehydration [30], or focused on standard postural movements (e.g., supine-to-stand or sit-to-stand) [30,31], leading to only modest results and a limited capacity for field applications. In a study by Cheuvront et al. [29], the difference between the average heart response of the final 10 s of 3 min of sitting and 1 min of standing provided fair discrimination of dehydration of 3% bodyweight loss. Owen et al. [30] aimed to estimate 2% bodyweight loss and achieved moderate accuracy by measuring the heart rate change 1 min after standing from a supine position. Though, participants in both studies performed the post-dehydration postural tests the day following their exercise session, which raises further uncertainty as to how such methods translate to a field application. In contrast, in this study, we leveraged the longitudinal heart rate response, monitored hydration immediately after exercise, and considered non-standard and shorter postural movements (e.g., toe-touches)—ultimately with a goal to develop an approach for field applications. We hypothesized that we could accurately detect exercise-induced dehydration using a combination of wearable technology that currently exists in the field and a varied set of postural movements, especially those more amenable to athletic environments (e.g., toe-touches).

## 2. Materials and Methods

### 2.1. Study Design and Setting

This study employed a controlled crossover design. The protocol was reviewed and approved by the University of Michigan Institutional Review Board (HUM00011582). Each participant provided written informed consent and the study was conducted in accordance with the Declaration of Helsinki.

### 2.2. Study Population

Physically active volunteers (10 males, 25.0 ± 6.6 years; 10 females, 27.8 ± 4.3 years) were recruited using the University of Michigan’s online recruitment tool (https://umhealthresearch.org, accessed on 6 May 2019) from May 2019 to February 2020. Using the area under the receiver operating curve (AUROC), a sample size calculation determined that 20 samples would be sufficient for detecting hydration status with a discriminative performance of at least AUROC = 0.74 (α = 0.05, β = 0.2) [32]. Given a randomly selected pair of positive and negative examples, the AUROC represents the probability of ranking the positive example higher than the negative example. For reference, an AUROC of 0.5 describes a model with discriminative performance no better than random chance, whereas an AUROC of 1.0 represents perfect discrimination. Healthy volunteers were screened for any history of cardiovascular, gastrointestinal, or musculoskeletal pathologies prior to enrollment. Volunteers were included if they were between ages 18 and 45, had a body mass index (BMI) below 30, and were not taking blood pressure or diuretic medication. Volunteers were screened for a minimum level of fitness and weekly activity; the inclusion criteria required an estimated VO_2_ max rating above the 70th percentile for adults of their age and sex [33]. We estimated VO_2_ based on a previously validated approach that relies on self-reported BMI, Perceived Functional Ability (PFA), the Physical Activity Rating Questionnaires (PAR-Q), and sex [34].

### 2.3. Study Interventions

Participants completed two experimental sessions scheduled 1–2 weeks apart within in a laboratory setting. To ensure euhydration upon arrival, participants were instructed to drink a prescribed amount of water before their session (7 mL/kg of bodyweight 4 h before the experiment, and 5 mL/kg of bodyweight 2 h before the experiment) [7]. Additionally, participants were instructed to fast by avoiding solid foods 2 h before the session. Participants voided upon arrival and a urine strip was used to measure urine specific gravity and verify hydration status. Nude bodyweight was then captured to the nearest 50 g using a Seca 703 (Hamburg, Germany) scale. Participants were provided a set of loose, moisture-wicking, athletic clothing.

During the first session, no fluids were provided during exercise. Using a Monark 928e (Vansbro, Sweden) cycle ergometer, participants warmed up for 5 min at 70 watts and subsequently exercised in 15-min bouts (with ~1 min between bouts) inside an enclosed, heated environment until they either (1) lost 2% of their initial nude bodyweight, or (2) completed 90 min of total exercise. Changes to bodyweight were repeatedly measured after each 15-min bout to track the percentage of bodyweight lost due to exercise. Participants toweled off and wore clothing during weight measurements until they lost roughly 1% of bodyweight, after which nude bodyweight measurements were taken until exercise ended. During the second session, participants exercised for the same number of bouts, and losses in bodyweight were measured and replenished with a prescribed amount of commercially available sports drink (Gatorade, Chicago, IL, USA). After drinking, participants’ bodyweights were measured again to verify that they attained their original bodyweights. Participants were asked to maintain a heart rate equivalent to 75% of their estimated maximum heart rate throughout exercise. Maximum heart rate for each participant was estimated by subtracting their age from 220 [35]. The heated environment consisted of a 6.5′ × 10′ walk-in greenhouse with a 1500-watt commercial feedback-controlled space heater (Patron™, Cheektowaga, NY, USA) set to 86 °F.

**Figure 1:**
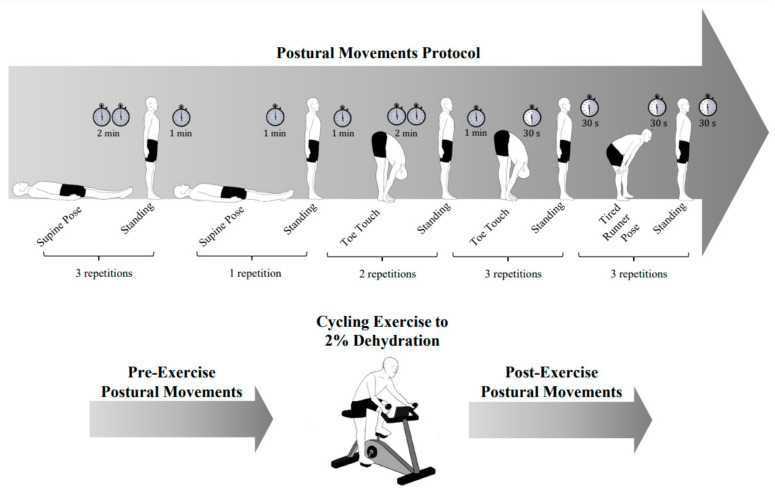
Scripted postural movements. Overall, 11 postural movements were performed before and after exercise (2% dehydration) during the dehydrated sessions with a varying number of repetitions. For the hydrated sessions, participants performed the postural movements following an equivalent amount of exercise needed to lose 2% bodyweight during the respective dehydrated sessions. The timing of the full postural movement sequence and the number of repetitions are shown in the top panel. The bottom panel shows the timing of the postural movements relative to the exercise component of the protocol. After transitioning to a standing position and completing a repetition, participants sat for 1 min.

Prior to and following the exercise portion of the study, participants performed a series of five scripted postural movements (i.e., “pre-exercise” and “post-exercise” movements) (Figure 1) outside of the heated environment. Each repetition of a postural movement consisted of two distinct positions, and a transition between the two positions. In order, they were:

- supine-to-stand test (2 min supine, 1 min standing; three repetitions),
- short supine-to-stand test (1 min supine, 1 min standing; one repetition),
- toe-touch stretch (2 min stretching, 1 min standing; two repetitions),
- short toe-touch stretch (30 s stretching, 30 s standing; three repetitions)
- “tired runner” pose (bending down with hands on knees, 30 s stretching, 30 s standing; three repetitions)

The supine-to-stand test was chosen because of its prominent use as a clinical tool for grossly screening dehydration [36]. We included the canonical version of the test, as well as a variation where we reduced the amount of time participants laid in the supine position. Other postural tests (i.e., toe-touch and “tired runner” pose) were included as they represented postures that are typical within an athletic setting.

For supine-to-stand movements, participants transitioned from a supine position to an upright position. For toe-touches, participants began by standing upright and then bent at the hip and reached for their toes. For the runner’s pose, participants leaned forward and rested their hands on their knees (we did not specify a specific angle and encouraged participants to assume a forward lean that felt natural). Between repetitions, participants sat on a chair for 1 min to allow their heart rate to return to the level prior to the postural movement. Afterwards, participants stood up and remained in the initial standing position for a few seconds before performing the next postural test. Participants completed the series of scripted postural movements in approximately 40 min. Throughout the scripted postural movements, participants were instrumented with a chest strap heart rate monitor (Polar H10, Polar, Kempele, Finland) to monitor heart rate and a wearable inertial measurement unit (Catapult OptimEye S5, Catapult, Melbourne, Australia) to measure postural orientation at 100 Hz.

### 2.4. Data Processing

We framed the hydration estimation task as a binary classification problem, where an accurate model would map the heart rate response during a postural movement to an estimate of the participant’s hydration status. Postural movements were labeled “dehydrated” if they were performed after exercise during the first session (no fluids). All other postural movements were labeled “euhydrated” given that they were performed either before exercise, or after an exercise session with fluid replenishment. To develop our model, we focused on the relative change in heart rate evoked by the transitions to standing during the postural movements.

**Figure 2:**
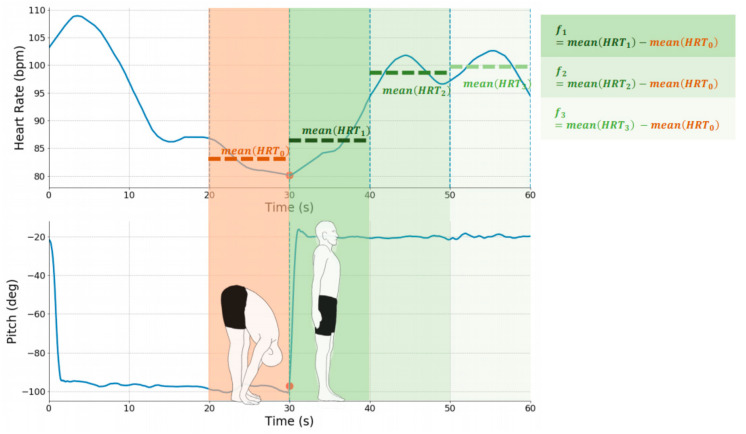
Feature extraction from a single postural movement. The heart rate response to the transition in the postural movement was calculated using differences between the mean heart rate for each segment and the mean pre-transition heart rate.

To compute relative change in heart rate, we started by smoothing the heart rate signal using a moving average (4-s window), and then divided the heart rate signal into a pre-transition response and a post-transition response. The transitions between postural positions were automatically detected based on the velocity of the pitch of the trunk during the postural movement. We then segmented the post-transition heart rate into three segments of equal length (e.g., divide 30 s of standing into three 10-s segments). As seen in Figure 2, the features used in our model were based on the difference between the average heart rate within each post-transition segment and the average pre-transition heart rate (i.e., average heart rate during 10 s prior to transition). This scheme effectively adjusted for inter-individual variances in resting heart rate and captured orthostatic effects rather than effects of exercise and recovery.

### 2.5. Model Training and Validation Scheme

To train and evaluate a model for assessing dehydration status based on extracted features, we iteratively split the data into training and testing sets. In each iteration, we reserved one participant’s postural movements for the test set, and the postural movements of all other participants were used to train the model. Compared to a random split, this approach estimated how the model would generalize to new participants. As postural movements that were labeled “dehydrated” only occurred after bouts of exercise, we focused our evaluation on post-exercise postural movements to ensure that the model was learning the effect of dehydration rather than exercise. For completeness, we also considered evaluating on pre-exercise and post-exercise postural movements and stratifying the analysis on male and female participants separately (see Supplementary Materials) [37,38].

To construct our model, we used L_2_ regularized logistic regression to learn a mapping from our computed features of heart rate to an estimate of hydration status. We considered other non-linear classifiers for the task of detecting hydration status as well but pursued the logistic regression model due to its superior cross-validation results, ease of training due to minimal hyperparameters, and its simplicity and interpretability (Supplementary Materials). We selected model hyperparameters based on the training data by maximizing the leave-one-out cross-validation AUROC [39].

Applied to each held-out participant, we evaluated the model’s ability to distinguish between hydrated and dehydrated examples based on the AUROC. We reported the AUROC averaged across participants, along with the interquartile range (IQR). To qualitatively evaluate the AUROC of our model, we referred to the descriptors outlined by Obuchowski et al. [40]. In addition to evaluating on all post-exercise postural movements, we evaluated on subsets of postural movements (e.g., toe touches only). Finally, we explored the importance of each feature by calculating Shapley values with respect to all post-exercise postural movements, using AUROC as the value function [41]. We reported the average and standard deviation of the Shapley values across all held-out participants. A larger Shapley value indicates a more important feature. To visualize these different segments, we computed and illustrated the heart rate responses for the post-exercise toe-touches between the hydrated and dehydrated sessions. We averaged the heart rate measurements at each sample (every 0.01 s) across all participants. Furthermore, we subtracted the average heart rate measured at the time of transition from the dehydrated and hydrated signal. Consequently, the signals were aligned at the time of transition, which facilitated fair comparisons between the post-transition responses. We specifically chose to present the post-exercise toe-touches to show the potential of shorter postural movements.

## 3. Results

### 3.1. Participant Characteristics

Participants lost 2.0% ± 0.3% of their bodyweight following exercise without replenishing fluids. Male participants weighed 75.4 ± 9.9 kg before exercise and 73.9 ± 9.7 kg after exercise (dehydrated sessions); female participants weighed 63.8 ± 5.5 kg before exercise and 62.5 ± 5.4 kg after exercise (dehydrated sessions). Self-reported PFA-1, PFA-2, and PAR-Q are shown in Table 1. Using a published regression formula [34], the average VO_2_ max for males and females were 53.4 ± 2.11 and 46.6 ± 3.61 mL·kg^−1^·min^−1^, respectively.

### 3.2. Model Performance

**Figure 3:**
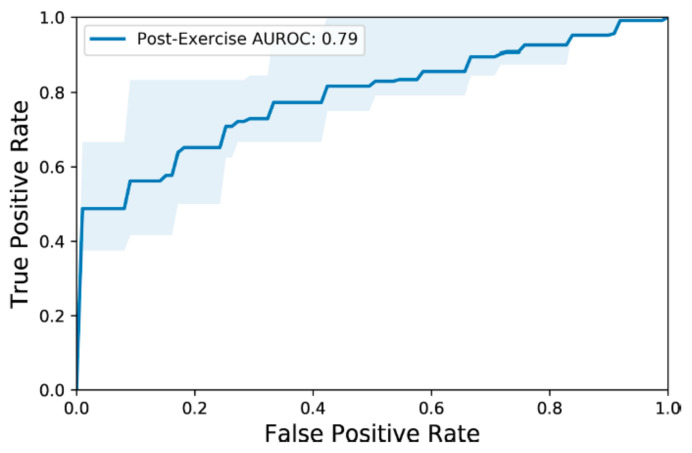
AUROC curve for the model when evaluating on post-exercise postural movements. The results were averaged across all participants as the test set. The shaded portion represents the IQR of the performance across the test participants.

We selected a regularization strength of 1 for each model based on the leave-one-out cross-validation performance. Our model achieved an average AUROC of 0.79 (IQR: 0.75, 0.91) when evaluating on post-exercise postural movements (Figure 3). Applied to the 2-min post-exercise supine-to-stand movements for the full population, performance improved (mean AUROC: 0.89, IQR: 0.89–1.0) (Table 2). Applied to the shorter 30 s toe-touches, the model achieved similarly strong discriminative performance (mean AUROC: 0.89, IQR: 0.89–1.0). In comparison, performance decreased slightly for the 2-min toe-touches (mean AUROC: 0.82, IQR: 0.81–1.0). For the 1-min supine-to-stand movement, the model achieved a mean AUROC of 0.79 (IQR: 1.0–1.0). Lastly, the 30-s “tired runner’s” pose achieved the lowest discriminative performance among the individual postural movements (mean AUROC: 0.77, IQR: 0.67–1.0).

### 3.3. Feature Importance

**Figure 4:**
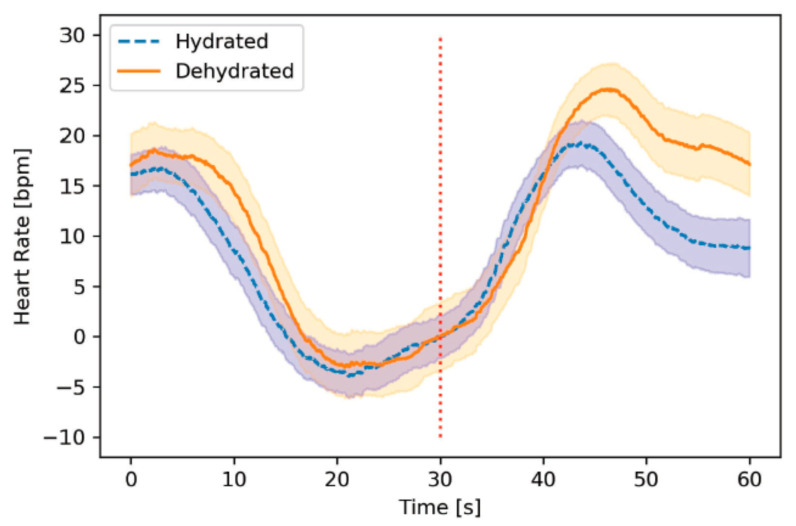
Average heart rate response to post-exercise toe-touches. The hydration session and dehydration session heart rate responses are shown, averaged across all participants and post-exercise trials. The vertical dashed line is halfway between the toe-touch and standing positions. Standard error is shown for each signal.

The Shapley values for the first, second, and third heart rate segments were 0.02 ± 0.05, 0.11 ± 0.07, and 0.15 ± 0.10, respectively. The heart rate responses during the first segment of the post-transition were similar during the hydrated and dehydrated sessions (Figure 4). The difference between the two heart rate responses was most pronounced during the final segment.

## 4. Discussion

Our results demonstrate that mild dehydration of at least 2% body weight loss can be detected noninvasively using readily available data from commercial wearables (i.e., heart rate and postural data), which is consistent with findings from prior laboratory-based studies that found orthostatic changes to be sensitive to levels of exercise-induced dehydration [29,30]. Moreover, accurate assessment did not necessarily require the longer clinical-based supine-to-stand movement. Instead, postural movements common in athletic settings, such as shorter toe-touches, could be used to detect mild dehydration.

At a level of 2% bodyweight loss, our model achieved between fair to high average AUROC for all the postural movements. Notably, the canonical 2-min supine-to-stand test and the 30-s toe-touches achieved the highest average AUROC (0.89). Although the 30-s “tired runner’s pose” had the lowest average AUROC (0.77, IQR: 0.67–1.00), the performance would still be considered fair [40]. The model’s performance on the shorter postural movements (i.e., toe-touches and “tired runner” pose) indicated that postural changes with movements commonly performed in athletic settings have the potential to be used for hydration assessments; these postural changes are likely to be seen when individuals are maximizing their recovery between repeated bouts of activity (e.g., during games/practices) [42]. The high average performance and tight inter-quartile ranges across participants also demonstrated the robustness of our model. In fact, the upper bound of the IQR for each postural test equaled 1.00, indicating a perfect classification for some individuals. When classifying all 24 post-exercise postural tests for a participant, our model achieved moderate performance (0.79, IQR: 0.75–0.91), demonstrating that data from wearables could be used for reliable predictions of mild dehydration.

Few studies have quantified the discriminative ability of the clinical orthostatic test, and even fewer studies have incorporated varied postural movements for estimating hydration status [29,30]. Previous work exploring the relationship between postural movements and post-transition heart rate responses have achieved only modest AUROCs. At an average dehydration of 2% bodyweight loss, Owen et al. [30] reported an AUROC of 0.66 for their supine-to-stand assessments. However, their dehydration protocol accounted for effects of exercise by assessing hydration two days after the exercise session. During this two-day period, participants followed a fluid restriction protocol. As a result, their participants reached a steady-state whereby orthostatic changes in heart rate may not have been as useful for discriminating hydration status. In comparison, we calculated relative changes in heart rate to address the immediate effects of exercise and obtained a higher AUROC (0.89), while also exploring different postural movements that are more commonly seen in athletic settings. Moreover, our assessment of hydration following the end of exercise (especially in heated environments) may be more valuable for early interventions of mild dehydration. At 3% bodyweight loss minimum, Cheuvront et al. [29] reported an AUROC of 0.67 using sit-to-stand movements and measurements of the absolute difference in the peak heart rate responses. Although our study assessed a lower percentage of bodyweight loss, our model still achieved a higher AUROC for different postural movements (i.e., 0.89 for the 30-s toe-touches). Our improved values of AUROC may be explained by our modified approach, which leveraged the longitudinal heart rate response to extract useful information and estimate dehydration. Furthermore, we tested immediately following exercise, which may have decreased the general variability in heart rate [43]. Cheuvront et al. [29] have cited heart rate variability contributing to the insensitivity of their approach. Ultimately, it was not possible to make direct comparisons to these methods as we assessed hydration status immediately following exercise.

The heart rate response closer to the end of the postural tests (which was 1 min after standing for supine-to-stand movements) influenced the model’s predictions more heavily, as indicated by the Shapley values. For comparison, some studies measured the change in participant heart rate 1 min after a supine-to-stand postural transition and similarly found a significant effect of dehydration [30,31]. For example, Owen et al. [30] reported a change of 26 ± 12 bpm when participants experienced 2% bodyweight loss, and 14 ± 8 bpm when hydrated. In an ultramarathon setting, Holtzhausen and Noakes [31] reported the change in heart rate 30–60 s after standing from supine (17 ± 8 bpm) to be significantly greater after the race than before (7 ± 9 bpm) the race. However, their study participants had a greater bodyweight loss percentage (4.6% ± 1.3%). The severe level of dehydration, intensity of the exercise, and environmental factors may have factored into the differences in reported values between the related studies. Overall, these studies’ findings are consistent with the results of our feature importance analysis; the heart rate response closer to the end of the postural tests provided the most useful information for classifying mild dehydration of 2% bodyweight loss.

When evaluating on all postural movements, the model achieved random or worse than random performance for two participants (9 and 17). We hypothesize that these differences are due to moderate changes in baseline bodyweight between experimental sessions (Table 1). Both subjects weighed more at the beginning of their dehydrated session than their hydrated session by 1.40 kg and 1.05 kg, respectively. Given that they lost 1.75 kg and 1.45 kg after exercise, their final post-exercise weight during the dehydrated sessions would have been relatively close to their baseline weight during their hydrated sessions, which may have led to similar orthostatic responses. Although we restricted fluid and food intake prior to the experiment, daily mass variability may have factored into the differences in baseline bodyweight. Our study did not account for participants’ daily mass variability, which may have introduced some uncertainty to bodyweight measurements as a proxy for hydration status. However, changes in daily mass have been estimated to be less than 1% in active men [44].

Laboratory-based detection methods that typically involve samples of bodily fluid, while accurate, are expensive and may be difficult to collect continuously in a fast-paced athletic context [10,14,15,23]. In contrast, clothed bodyweight measurements provide one of the quickest and most accessible assessments of hydration with minimal equipment in the field (e.g., a scale situated on the sidelines). However, if nude bodyweight measurements are not feasible, excess sweat in the clothing and on the athlete should be minimized to obtain the most accurate and precise measurements [45]. In our laboratory-based study, we used nude bodyweight measurements throughout a cycling exercise to train a model and make predictions of dehydration. As such, our model learned how to weight features based on accurate measurements of nude bodyweight, which improved the reliability of the model predictions. Therefore, our method may potentially complement clothed bodyweight measurements by leveraging increasingly available data from wearable sensors. As recommended by Barley et al. [23], combining our approach and gross bodyweight measurements may therefore lead to an increase in overall reliability. In a practical setting, our method may inform athletes when they are approaching mild levels of dehydration and enable early interventions, such as taking additional informed measurements of bodyweight, before potentially reaching severe levels of dehydration.

Similar to the capabilities of biometric sensors, our approach has the potential to continuously gather samples and track hydration status over time, but our approach requires an individual to make distinct postural movements. On the other hand, our approach does not require specialized sensors. Other methods for estimating dehydration including serum analysis (i.e., blood work), urinalysis, saliva analysis, thirst, and dilution techniques [10,23] vary in cost, time-to-administer, accuracy, portability, and invasiveness.

Our study was not without limitations. First, cycling in a heated environment was used to dehydrate participants; it is unclear how our results might generalize to other methods of dehydration, especially passive approaches (e.g., heat exposure) [17,46,47]. Second, we relied on bodyweight to measure the level of dehydration. While blood sample analysis may be more accurate it is more difficult to obtain. Third, we designed the study such that the dehydrated session preceded the hydrated session in the case that, if participants dropped out after the first session, we would still have relevant data on dehydrated individuals. As a result, this could have caused habituation to the protocol, particularly for individuals with minimal cycling experience. However, we only included participants above an estimated level of fitness with no history of cardiovascular disease (though fitness was not directly measured). Fourth, postural movements were performed in the same order each time. Thus, heart rate following exercise may have recovered substantially during the later postural movements (e.g., “tired runner’s pose”). Additionally, participants sat between repetitions, which may have affected heart rate responses due to the dynamic shift in body fluids. Finally, fluids were replenished periodically throughout the experiment as well, meaning that fluids administered near the end of the exercise session may not have been fully absorbed by the time participants performed the postural tests.

We note that some of these limitations have been present for many past laboratory-based dehydration studies. For example, in laboratory-based settings, ecologically valid exercise conditions can be difficult to replicate, which raises some uncertainty when studying the effect of hydration on physical performance [9,48]. Despite these limitations, our study provided a meaningful step towards potentially automating noninvasive measurements of dehydration, which may eventually improve hydration practices and health monitoring. In addition to reducing the risk of heat-related injuries, proper hydration may also maintain physical performance during activity [4,49,50,51]. In its current form, our method may not be directly applicable to natural field settings. However, this work illustrated the efficacy of using increasingly readily available data from wearable sensors for detecting hydration status, while also using shorter and more diverse postural movements than previously considered.

## 5. Conclusions

Overall, our method produced reliable and accurate predictions of mild dehydration (2% bodyweight loss) 30 s after a postural transition using wearable sensors to measure heart rate and postural orientation. Moreover, the approach required minimal, noninvasive, commonly used wearable sensors. In future implementations, such an approach would complement existing bodyweight measurements, and potentially allow earlier interventions of dehydration. Future work should incorporate more ecological exercise conditions to validate the efficacy of such an approach in natural field settings.

## Figures and Tables

**Table 1:** Characteristics for each participant.

ID	Age [yrs.]	Sex	Height [cm]	Initial BW, DEH [kg]	Bodyweight Lost [%]	Initial BW, HYD [kg]	BMI [kg∙m^−2^]	PFA [1, 2]	PAR-Q	VO2max [ml∙kg^−1^∙min^−1^]
1	23	M	182	85.60	1.52	88.50	25.8	11, 9	9	51.7
2	25	M	195	98.10	2.14	98.40	25.3	11, 10	7	51.4
3	27	F	165	66.10	1.21	65.00	23.8	11, 9	7	51.9
4	27	M	172	66.40	1.58	66.30	22.9	12, 9	7	54.1
5	23	M	182	70.45	2.20	70.60	21.7	13, 11	8	57.3
6	19	M	163	70.85	1.98	70.50	26.6	11, 10	7	50.4
7	27	F	178	65.95	2.43	65.00	20.8	11, 10	7	48.1
8	25	M	165	76.50	2.09	76.15	25.5	11, 11	9	53.4
9	42	M	195	76.80	2.28	75.75	22.1	11, 11	8	55.5
10	24	F	175	75.80	2.31	75.65	24.4	10, 8	7	42.9
11	27	F	155	59.45	1.93	59.35	25.3	9, 9	7	42.2
12	23	F	167	66.50	2.03	67.50	24.2	11, 10	7	45.3
13	28	F	170	59.00	2.03	59.35	20.8	12, 12	8	51.0
14	38	F	160	58.55	2.04	57.60	22.7	9, 7	7	42.8
15	18	M	180	75.20	2.53	75.10	24.0	11, 9	7	51.8
16	30	F	170	59.95	1.83	59.25	20.4	9, 9	7	46.2
17	26	M	178	67.35	2.15	65.95	20.8	11, 9	7	54.4
18	22	M	163	66.75	1.95	68.25	24.9	12, 11	8	53.9
19	30	F	170	67.10	2.01	67.10	23.8	11, 9	7	44.9
20	24	F	170	59.70	2.09	59.80	19.6	11, 11	8	50.5

Abbreviations: BMI = Body Mass Index, BW = Nude Bodyweight, PFA-1 = Perceived Functional Ability First Rating (assesses ability to run 1 mile), PFA-2 = Perceived Functional Ability Second Rating (assesses ability to run 3 miles), PAR-Q= Physical Activity Readiness Questionnaire, DEH = Dehydrated Session, HYD = Hydrated Session.

**Table 2:** Distribution of classification performance when evaluating on specific postural movements post-exercise.

Evaluated Postural Movements	Mean AUROC (IQR)
All	0.79 (0.75, 0.91)
2-min Supine-to-Stand	0.89 (0.89, 1.00)
1-min Supine-to-Stand	0.79 (1.00, 1.00)
2-min Toe-Touch	0.82 (0.81, 1.00)
30-s Toe-Touch	0.89 (0.89, 1.00)
30-s Runner’s Pose	0.77 (0.67, 1.00)

Abbreviations: AUROC = Area Under Receiver-Operating-Curve, IQR = Interquartile Range.

## Data Availability

The data presented in this study are available on request from the corresponding author. The data are not publicly available due to ongoing analysis.

## Supplementary Materials

The following are available online at https://www.mdpi.com/article/10.3390/s21134469/s1, Supplementary data S1: Evaluation of model on all postural movements, stratified by participants’ sex, Table S1: Distribution of classification performance when evaluating on specific postural movements, Figure S1: AUROC curve for the model evaluated for each evaluation when including pre-exercise movements, Figure S2: Average heart rate response all toe-touches, Table S2: Results stratified based on the sex of the held-out test individual.
