# Supplementary material for: Noninvasive Estimation of Hydration Status in Athletes Using Wearable Sensors and a Data-Driven Approach Based on Orthostatic Changes
Source: Sensors (Basel). 2021 Jun 29;21(13):4469. doi: 10.3390/s21134469 (PMC8271939; doi:10.3390/s21134469)
Supplement: Supplementary file 1 [file sensors-21-04469-s001.zip › S1.pdf]

Additional Evaluations

Throughout the main text, we focused evaluation on postural movements that occurred after exercise. Here, we describe the evaluation of all postural movements.

Applied to each held-out participant, we evaluated the model's ability to distinguish between hydrated and dehydrated examples based on the AUROC. We report the AUROC averaged across all participants, along with the interquartile range (IQR). As described in the main text, our model achieved an average AUROC of 0.82 (IQR: 0.75-0.91) when evaluating on all postural movements (Figure S1). Overall, these evaluations followed the same trend as when pre-exercise postural movements were not included in the evaluation set. However, the resulting performance was slightly better, as expected due to pre-exercise heart rate responses generally being lower than heart rate responses after exercise. Similar to when we evaluated the model on only post-exercise movements, the performance was even better when evaluating on only the 2-min clinical supine-to-stand test (mean AUROC: 0.92, IQR: 0.85-1.0) and the 30-s toe-touches (mean AUROC: 0.88, IQR: 0.78-1.0) (Table S1).

Table S1. Distribution of classification performance when evaluating on specific postural movements.

Evaluated Postural Movements	Mean AUROC (IQR)
All	0.82 (0.75, 0.91)
2-min Supine-to-Stand	0.92 (0.85, 1.00)
1-min Supine-to-Stand	0.91 (0.92, 1.00)
2-min Toe-Touch	0.85 (0.79, 1.00)
30-s Toe-Touch	0.88 (0.78, 1.00)
30-s Runner Pose	0.79 (0.63, 1.00)

When evaluating on all pre-exercise and post-exercise postural movements, the model had high discriminative performance, especially when evaluating on shorter 30-s toe-touches.

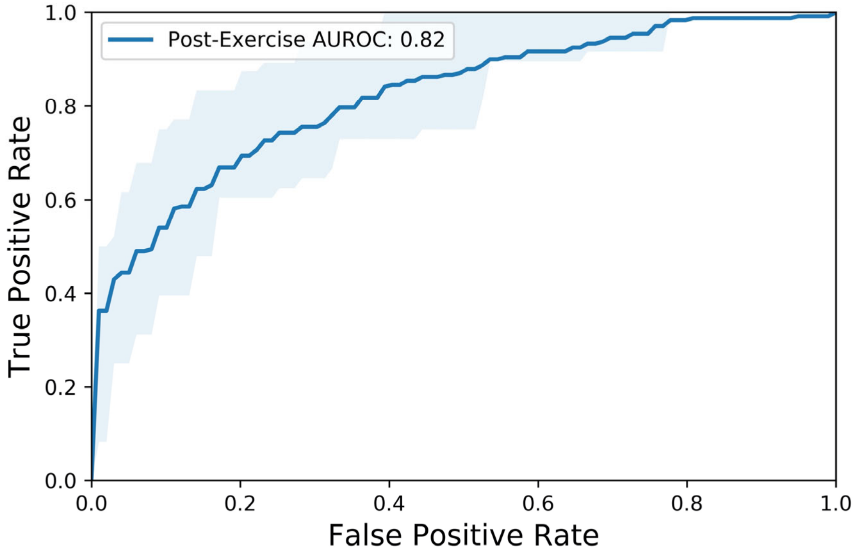

Fig. S1 AUROC curve for the model when including pre-exercise movements. The results were averaged across all participants as the test set. The shaded portion represents the IQR of the performance across the test participants.

We also considered the measures of feature importance of the logistic regression model when evaluating on all postural movements. Using the AUROC as a measure of importance, the Shapley values of the average post-transition heart rate over all participants were 0.02 ± 0.05 for the first segment, 0.11 ± 0.05 for the second segment, and 0.20 ± 0.08 for the third segment. Visual inspection of the differences in heart rate response to all toe-touches (Figure S2) led to similar conclusions as when excluding pre-exercise postural movements. The difference in heart rate between hydrated and dehydrated toe-touches was most pronounced during the final two-thirds of the response to the transition in the postural movement.

Next, we considered other non-linear classifiers for the task of detecting hydration status. For completeness, we reported the results of the tree-based non-linear model based on a random forest to show the strong performance of the logistic regression model. Here we reported the AUROC averaged across all participants, along

with the interquartile range (IQR). When evaluating on just post-exercise movements, the random forest achieved an AUROC of 0.68 (0.57, 0.86). When evaluating on all pre-exercise and post-exercise movements, the random forest achieved an AUROC of 0.73 (0.62, 0.85). In contrast, the L2-regularized logistic regression model achieved an AUROC of 0.79 (0.75, 0.91) when evaluating on post-exercise movements only, and an AUROC of 0.82 (0.75, 0.91) when evaluating on all postural movements. We hypothesized that the strong performance of the linear model and the poor performance of the non-linear model were due to the limited number of features and samples.

Finally, we considered the model's performance when stratifying based on the sex of the held-out test individual for each set of postural movements used for evaluation (Table S2). In general, the model performed better when evaluating on females. We hypothesized that this result was due to variations in our study population. First, the poor performance for males was largely driven by the two individuals for whom the model achieved random or worse than random performance. These two individuals had significant changes in baseline bodyweight between sessions, which caused the average model performance to deteriorate for males. Moreover, the variability of height and weight among participants was much greater among male than female participants, which may have negatively influenced the orthostatic responses to the postural movements. The standard deviations of height and weight for male participants were 11.9 cm and 9.9 kg, respectively; for females, these values were 6.7 cm and 5.5 kg, respectively.

Another possible explanation for why the model achieved higher performance for females than males may have been because of the effect of sex on orthostatic

responses. Women are generally less efficient in their compensatory response to orthostatic changes (i.e., raising their heart rate) due to a more active parasympathetic system and a lower center of gravity [48]. One study suggested that women have greater orthostatic intolerance due to a smaller stroke volume (and therefore a weaker compensatory response), especially when they are passively dehydrated from a diuretic [49].

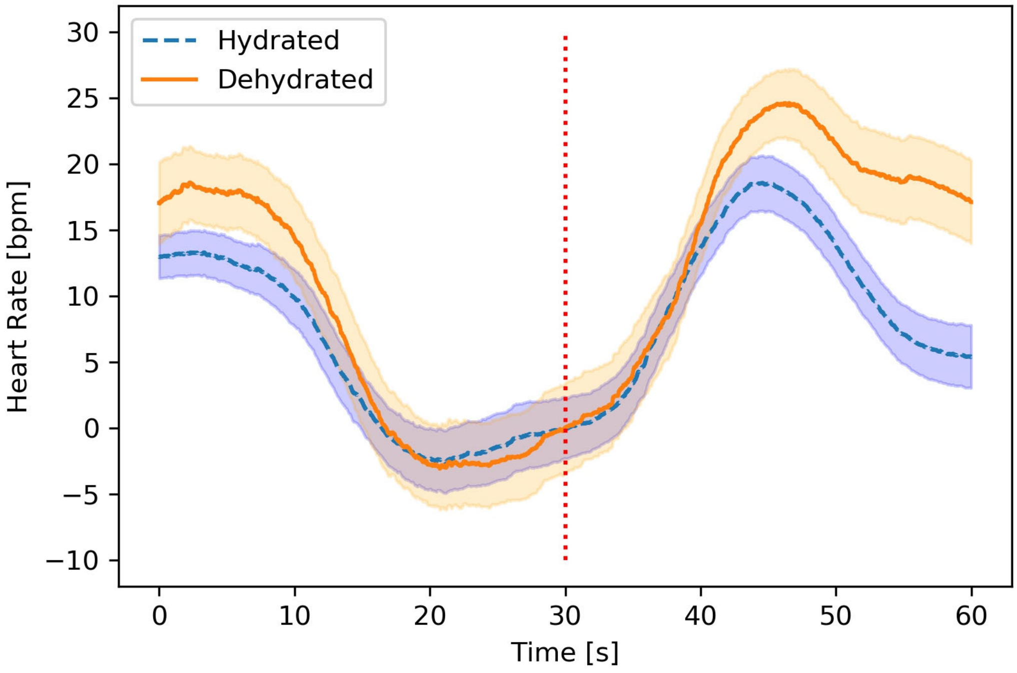

Fig. S2 Average heart rate response for all toe-touches. The heart rate responses are shown for a hydrated session and a dehydrated session separately, averaged across all participants and trials. The vertical dashed line is halfway between the toe-touch and standing positions. Standard error is shown for each signal.

Table S2. Results stratified based on the sex of the held-out test individual.

Evaluation Set	Mean AUROC (IQR) for Males	Mean AUROC (IQR) for Females
All Postural Movements	0.78 (0.69, 0.86)	0.87 (0.82, 0.98)
Post-Exercise Movements	0.69 (0.64, 0.80)	0.90 (0.82, 1.00)

Regardless of the evaluation set, the model consistently performed better when evaluating on females than when evaluating on males.

References

37. Cheng, Y.C.; Vyas, A.; Hymen, E.; Perlmuter, L.C. Gender differences in orthostatic hypotension. *Am. J. Med. Sci.* **2011**, *342*, 221–225, doi:10.1097/MAJ.0b013e318208752b.

38. Fu, Q.; Witkowski, S.; Okazaki, K.; Levine, B.D. Effects of gender and hypovolemia on sympathetic neural responses to orthostatic stress. *Am. J. Physiol. - Regul. Integr. Comp. Physiol.* **2005**, *289*, 109–116, doi:10.1152/ajpregu.00013.2005.
